# The DNA Damage Response in Fully Grown Mammalian Oocytes

**DOI:** 10.3390/cells11050798

**Published:** 2022-02-24

**Authors:** Alexandros Pailas, Konstantina Niaka, Chrysoula Zorzompokou, Petros Marangos

**Affiliations:** 1 Department of Biological Applications and Technology, School of Health Sciences, Institute of Biosciences, University Research Centre, University of Ioannina, 45110 Ioannina, Greece; xanderpailas@gmail.com (A.P.); niakonina@hotmail.com (K.N.); chrisaz212@gmail.com (C.Z.); 2 Biomedical Research Institute, Foundation for Research and Technology, University of Ioannina Campus, 45115 Ioannina, Greece

**Keywords:** oocyte, DNA damage response, checkpoints, DNA repair

## Abstract

DNA damage in cells can occur physiologically or may be induced by exogenous factors. Genotoxic damage may cause cancer, ageing, serious developmental diseases and anomalies. If the damage occurs in the germline, it can potentially lead to infertility or chromosomal and genetic aberrations in the developing embryo. Mammalian oocytes, the female germ cells, are produced before birth, remaining arrested at the prophase stage of meiosis over a long period of time. During this extensive state of arrest the oocyte may be exposed to different DNA-damaging insults for months, years or even decades. Therefore, it is of great importance to understand how these cells respond to DNA damage. In this review, we summarize the most recent developments in the understanding of the DNA damage response mechanisms that function in fully grown mammalian oocytes.

## 1. The DNA Damage Response in Somatic Cells

DNA damage is a form of cellular stress defined as any kind of alteration in the DNA that disrupts its major functions (replication and transcription) [1]. Cells possess DNA damage response (DDR) mechanisms in order to counter DNA lesions. The role of the DDR is to detect damaged DNA and signal towards its repair. The DNA damage response consists of a variety of proteins that can be categorized into sensors, mediators, transducers and effectors (Table 1). The cellular responses include cell cycle arrest, chromatin remodeling, repair of the damage or apoptosis/senescence [2,3]. Once the DDR detects a DNA lesion, a DNA damage checkpoint may be launched which will induce cell cycle arrest until the damage has been repaired through the DNA damage repair machinery of the DDR. If the damage is extensive and unrepairable, the DDR may induce cell death through apoptosis.

DNA lesions can be programmed or spontaneous. Programmed DNA lesions are part of physiological cellular processes, such as the meiotic homologous recombination of gametogenesis, or the immune response to pathogens [2].

Spontaneous DNA lesions are unprogrammed, potentially harmful to the cell and can be caused by endogenous and exogenous factors. Endogenous DNA damage occurs naturally during DNA metabolism. Endogenous DNA damage may be the result of spontaneous base deamination, spontaneous creation of abasic (AP) sites, replication errors and mismatches, topoisomerase enzymes and oxidation stress and reactive oxygen species (ROS) created physiologically during cellular metabolic processes. Exogenous DNA damage is caused by environmental factors which can be physical or chemical agents. Physical agents include ultraviolet light or ionizing radiation. The most common chemical agents that cause DNA damage are alkylating agents, topoisomerase inhibitors, aromatic amines and polycyclic aromatic hydrocarbons (PAHs) [5,6].

**Figure 1:**
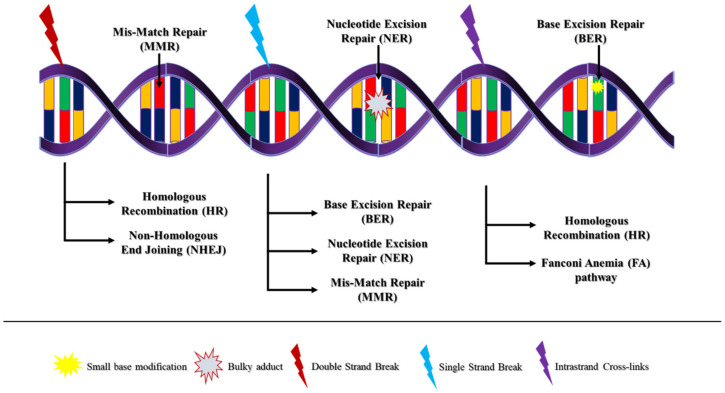
The main repair pathways utilized by the eukaryotic cell.

The most common types of DNA lesions are base mismatches, chemical modifications of the DNA bases that do not affect the helix, bulky base alterations that distort the double-helix structure, single-strand breaks (SSBs), intrastrand crosslinks (ICLs) and double-strand breaks (DSBs). Cells have evolved several mechanisms to repair these lesions in order to maintain genome integrity [6,7]. More characteristically, DSBs are repaired by homologous recombination (HR) or nonhomologous end-joining (NHEJ), while SSBs are repaired through base excision repair, nucleotide excision repair and mismatch repair (Figure 1).

In cycling cells, in order to prevent progression through the cell cycle in the presence of unrepaired DNA damage, the cell cycle is arrested by the activation of DNA damage checkpoints (DDCs). Recently however, a model based on brakes has been suggested, instead of the classical checkpoint-based DDR arrest model. According to this approach, the term checkpoint can be confusing since it traditionally refers to a specific point of the cell cycle where certain conditions are checked. However, the components responsible for the DDR-dependent halting of the cell cycle are present and may become active throughout the cell cycle phases and not at a specific cell cycle point [8].

The main aim of these checkpoints is to delay or halt the cell cycle by keeping cyclin-CDK complexes inactive. DDCs are mostly enabled at the transitions between different cell cycle phases, such as the G1/S and G2/M transitions, or during S phase [2,4].

At the G1/S checkpoint, depending on the type of damage, DSB or SSB, ATM (*Ataxia*-telangiectasia mutated) kinase or ATR (*Ataxia*-telangiectasia mutated and Rad3-related) kinase are initially activated, respectively. These kinases phosphorylate and activate checkpoint kinases: ATM phosphorylates Chk2, while ATR phosphorylates Chk1. Both Chk1 and Chk2 phosphorylate and inhibit Cdc25A, a phosphatase that is essential for the activation of Cyclin E-CDK2. The Cyclin E-CDK2 complex is responsible for the phosphorylation and activation of Cdc45, a factor crucial for the initiation of DNA replication in S phase. In addition, both ATM and ATR phosphorylate and stabilize the transcription factor p53. p53 leads to the activation of p21, a CDK2 inhibitor and CDK4 stabilizer. These processes will arrest cycling cells at the G1 phase of the cell cycle until the damage is repaired [2,3,4]. 

The main structure that activates the intra-S checkpoint is single-stranded DNA (ssDNA) coated with the RPA protein. This structure is recognized by ATR-interacting protein (ATRIP) translocating ATR to the damaged sites. In addition, the checkpoint sliding-clamp complex, Rad9/Rad1/Hus1 (9-1-1), which provides a scaffold for the assembly of the DNA damage recognition complex, and the DNA Topoisomerase II Binding Protein 1 (TopBP1) lead to the activation of ATR. ATR then phosphorylates Chk1 which subsequently phosphorylates factors that lead to a delay in replication firing and progression of DNA synthesis. Other types of lesions such as ICLs and DSBs produce ssDNA while they are being processed, and may, therefore, enable an ATR-dependent checkpoint [4,9].

As in the G1/S checkpoint, the G2/M checkpoint is launched by the activation of ATM or ATR in response to DSBs or SSBs, respectively. ATM and ATR activate Chk2 and Chk1 which subsequently inactivate the Cdc25A phosphatase and phosphorylate and activate the Wee1 kinase. As a result, the kinase complex responsible for M-phase entry, Cyclin B-CDK1, remains inactive and the cell cycle is arrested at G2 until the lesion is repaired [2].

## 2. Mammalian Oocyte Development and Oocyte Maturation

During embryonic life in female mammals, the entire pool of germ cells enters meiosis, and following homologous recombination, the oocytes become arrested at meiotic prophase. The oocytes stored in the primordial pool of ovarian follicles remain arrested at prophase due mainly to the absence of expression, at the protein or mRNA level, of the factors that are necessary for meiotic resumption [10]. From puberty, one or more oocytes enter a hormonally induced growth phase, during which they build a reserve of mRNAs and proteins which will be used in the meiotic process, but also during the early stages of embryonic development. The resulting fully grown oocytes have acquired a substantial pool of mRNAs and proteins that allows them to possess meiotic competence, which is the ability to resume and complete meiosis. These oocytes have a large nucleus (Germinal Vesicle—GV) with highly condensed chromatin that surrounds the nucleolus and are transcriptionally silenced.

As in the case of the G2 DNA damage checkpoint, during fully grown oocyte prophase arrest, the Cyclin B-CDK1 kinase complex is maintained inactive and thus entry into M-phase is inhibited. Considering the fact that the launch of the G2 DDC is mostly transcription-independent, the G2 DDC significantly resembles the prophase arrest seen in fully grown oocytes [11]. In both cases, the state of arrest depends largely on a series of kinase-dependent phosphorylations. However, unlike the G2 DDC, prophase arrest is not imposed by ATM/ATR and Chk1/Chk2. During prophase arrest, the high levels of cytoplasmic cAMP cause the activation of Protein Kinase A (PKA). PKA phosphorylates mainly the Wee1 kinase and Cdc25 phosphatases. As a result, Wee1 becomes activated and Cdc25 is silenced. This leads to the inhibitory phosphorylation of Cyclin B-CDK1 by Wee1, while the inactive Cdc25 cannot remove the inhibitory phosphates of the Cyclin B-CDK1 complex [12,13].

**Figure 2:**
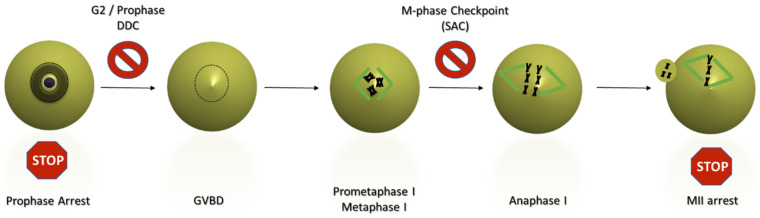
States of arrest and checkpoints during mammalian oocyte maturation. The mammalian oocyte enters prophase arrest in embryonic life. Resumption of meiosis can occur after the onset of puberty. Following ovarian exposure to LH, the oocyte resumes meiosis, undergoes germinal vesicle breakdown (GVBD) and enters the first meiotic M-phase. After a lengthy prometahase I during which the first meiotic spindle is formed, homologous chromosome pairs align at the spindle equator. The Spindle Assembly Checkpoint (SAC) inhibits chromosome disjunction until all the chromosomes are properly attached to spindle microtubules from opposite spindle poles and under tension from pulling forces directed towards the poles. Chromosome segregation leads to an asymmetric meiotic division and immediate entry into meiosis II, where the oocyte arrests at metaphase II (MII) awaiting fertilization. Severe DNA damage imposed during prophase arrest launches the G2/prophase DNA damage checkpoint (DDC) which inhibits the resumption of meiosis. A damaged oocytes that slips through the DDC may become arrested during the first meiotic M-phase due to the activation of the SAC.

Following the surge in the luteinizing hormone (LH), fully grown oocytes exit the state of prophase arrest and resume meiosis. Resumption of meiosis involves the inactivation of PKA and Wee1 kinases and the activation of Cdc25 phosphatases. As a result, Cyclin B-CDK1 is activated, leading to GV breakdown (GVBD) and entry into a lengthy meiotic M-phase (MI), during which the first meiotic microtubule spindle is formed. As soon as all the homologous chromosome pairs attach to spindle microtubules and align at the metaphase plate, Cyclin B is degraded through the actions of the E3 ubiquitin ligase Anaphase Promoting Complex/Cyclosome (APC/C). This leads to Cyclin B-CDK1 inactivation and the subsequent segregation of homologous chromosomes [12]. The first meiotic division is immediately followed by entry into the second meiotic M-phase, where the oocyte is arrested at metaphase II (MII) awaiting fertilization. The process from meiotic resumption to MII arrest is known as oocyte maturation (Figure 2). 

## 3. The G2/Prophase DNA Damage Checkpoint in Mammalian Oocytes

Studies on the effects of DNA damage on the meiotic cell cycle of mammalian oocytes were first performed in the 1990s. In these early reports, GV-stage oocytes were subjected to ultraviolet irradiation (UV-A or UV-C). UV irradiation did not seem to affect GVBD but affected M-phase progression and completion of MI [14].

Studies during the past decade have shed more light at the mechanistic level on how the oocyte cell cycle is regulated following DNA damage. DSBs are recognized by the presence of the phosphorylated form of H2AX, γH2AX [15]. Detection of DSBs and the accumulation of γH2AX is facilitated by the DNA damage sensor complex Mre11-Rad50-NBS1 (MRN), and specifically meiotic recombination 11 (Mre11) [16]. DSBs induce different responses depending on their severity. High levels of DSB-producing damage in fully grown mouse oocytes following the use of the Topoisomerase-II inhibitor etoposide or bleomycin leads to impairment of meiotic resumption and oocytes remaining arrested at prophase (GV stage) [17,18]. The maintenance of this arrest is mediated by ATM which phosphorylates Chk1, and Chk1 inhibits, through phosphorylation, the phosphatase Cdc25B [18]. G2/prophase arrest also appears in human oocytes treated with high levels of genotoxic agents, such as etoposide [19].

**Figure 3:**
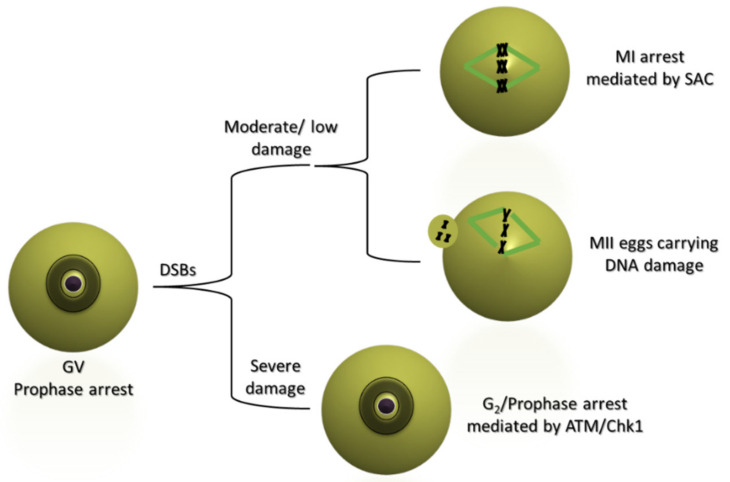
The DNA damage checkpoints in mammalian oocytes. DSBs that lead to severe DNA damage in prophase-arrested oocytes (GVs) launch an ATM/Chk1-dependent checkpoint which maintains prophase arrest. The G2/prophase checkpoint is not sensitive enough to detect moderate or low levels of damage. Potentially, prolonged GV culture or coculture with cumulus cells may maintain the state of arrest. Oocytes that enter the first meiotic M-phase in the presence of DNA damage either arrest in M-phase due to actions of the Spindle Assembly Checkpoint (SAC) or complete oocyte maturation and arrest at the second meiotic metaphase (MII) in the presence of DNA damage.

Strikingly, lower levels of damage, induced by low doses of etoposide, neocarzinostatin or bleomycin, have no or minimal effect on meiotic resumption and maturation [17,18,19,20]; mouse and human oocytes resume meiosis and undergo GVBD. Furthermore, in porcine oocytes, etoposide and bleomycin caused DSBs that did not inhibit GVBD [21,22]. This inability to establish a G2/prophase arrest is primarily attributed to the insufficient activation of the ATM/Chk1 pathway [18,21]. Recently, it was proposed that the inability of mouse oocytes to establish robust G2/prophase arrest is attributed to the persistent activity of the wild-type p53-induced phosphatase 1 (Wip1), which may hinder the phosphorylation and therefore activation of ATM. When Wip1 is pharmacologically inhibited at the GV stage, ATM activation levels rise enabling the establishment of a DDC in the presence of DNA damage which leads to the inhibition of GVBD and the subsequent resumption of meiosis [23]. Another possibility is that mammalian oocytes are delayed in their response to DNA damage. A recent study suggests that oocytes launch a G2/prophase DDC several hours (>20 h) after the damage occurs [24]. The authors show that the DDC is established through the increased activity of the APC/C. APC/C then mediates the proteolysis of cyclin B. This increased APC/C activity is due to increased activity of the protein phosphatase Cdc14B and decreased activity of the APC/C inhibitor Emi1. Furthermore, other reports suggest that the oocyte G2/prophase DDC becomes more robust when the oocyte is enclosed within the cumulus attached to follicular cumulus cells (cumulus enclosed oocyte—COC). This may be the result of a rise in the production of cAMP in cumulus cells in response to DNA damage, which can then pass to the oocyte through the gap junctions that link the oocyte with the cumulus cells [25]. In general, mammalian fully grown oocytes possess a weak or delayed G2/prophase DDC that is primarily activated under conditions of severely damaged DNA (Figure 3).

## 4. The M-Phase Checkpoint in Response to DNA Damage

The extent and means of induction of DNA damage may determine whether resumption of meiosis I will lead to the first meiotic division, which is marked by the extrusion of the first polar body (Pb1). DSBs induced by zeocin, bleomycin or laser microbeams can lead to completion of meiosis in mammalian oocytes [17], although extensive DNA damage may lead to the delay of Pb1 extrusion or even cell cycle arrest during MI [22,26].

M-phase arrest in MI following DNA damage does not seem to be caused by the activation of traditional DDC regulators. It has been shown that ATM or ATR inhibition does not alleviate DNA damage-induced MI arrest [27,28]. Instead, MI arrest in response to DNA damage is caused by the activation of the Spindle Assembly Checkpoint (SAC), the master mechanism responsible for the surveillance of M-phase and the correct alignment and binding of chromosomes to the M-phase microtubule spindle. When chromosomes are misaligned or not attached to spindle microtubules, the SAC is activated and SAC components are localized at the chromosome kinetochores, at the sites of centromeric chromatin, where they inhibit the APC/C, and therefore Cyclin B degradation and M-phase exit (Figure 2) [29].

SAC activity has been observed to be elevated in MI mammalian oocytes following DNA damage performed at the GV stage [17,26,28,30]. SAC activation might be enabled by DNA damage-induced chromosome fragmentation, although DNA damage does not seem to induce substantial disruption of chromosome biorientation and spindle formation [30]. The importance of the SAC for establishing MI arrest in response to DNA damage is nicely demonstrated by experiments where the SAC is disrupted: inhibition or downregulation of Mps1 or Mad2, key components of the SAC complex, alleviate the DNA damage-induced MI arrest [28,30]. In mouse oocytes subjected to DNA-damaging agents during MI, the recruitment of SAC components on the kinetochores occurs within minutes from the DNA damaging insult.

SAC-induced MI arrest in response to DNA damage has also been shown to depend on SAC regulators, such as Aurora kinases and Haspin, which are responsible for the recruitment of SAC components to the kinetochores [27]. Although etoposide-induced DSBs may be present in the full extent of the chromosome, including both the centromeres and the chromosomal arms, the recruitment of SAC components occurs only on the kinetochores [27]. However, there is no obvious loss in kinetochore-attached microtubule fibers (k-fibers), or tension across the bivalents. In a separate report, Doxorubicin-induced DSBs also enable an SAC-dependent checkpoint and MI arrest; however, in this case, k-fibers are affected [31]. These differences possibly represent the different mechanisms of action of the genotoxic agents, or the different potencies of the drug concentrations used.

It must be noted that the establishment of a DNA-damage-induced checkpoint at MI is age-dependent. It has been shown that in aged mouse oocytes, DNA damage cannot cause MI arrest because of poor recruitment of SAC components at chromosomal kinetochores [28]. Therefore, in females of advanced reproductive age, DNA damage may impose severe chromosomal anomalies, which are detrimental for embryonic development.

Although DNA damage can launch an SAC response in MI, the DNA damage surveillance mechanisms might not be very sensitive at this stage, and on many occasions, oocytes with persistent DNA damage may complete MI and reach MII (Figure 3). Characteristically, human oocytes do not seem to arrest at MI in response to DNA damage [19]. This explains why MII oocytes subjected to DNA damage at the GV stage show a degree of chromosomal fragmentation [26,28,30] or abnormal chromosome metaphase II plates and spindles [31]. Chromosomal fragmentation in MII oocytes is also witnessed after inducing DNA damage in vivo in mice [26]. It is possible that DNA-damage-induced establishment of the SAC at MI can only occur if the damage occurs at the kinetochore region of centromeric DNA. The inability of the SAC to detect chromosomal arm DNA damage may explain the weak MI DNA checkpoint response. This weakness might explain why cytoplasmic chromosomal fragments that result from chromosomal arm DSBs are seen in MII oocytes subjected to DNA damage from the GV stage.

In addition, although the SAC is activated in MII, in response to MII spindle disruption or chromosome misalignment, DNA damage does not seem to launch a SAC-dependent checkpoint in MII oocytes. This was determined by the fact that there is no significant recruitment of SAC components on the MII kinetochores following DNA damage [27].

## 5. DNA Repair in Mammalian Oocytes

The purpose for the establishment of a DDC is to arrest the cell cycle while the cell repairs DNA lesions. Although there are strong indications that DNA repair occurs in oocytes, the processes, the clear mechanisms, the efficacy and speed of repair in oocytes compared to somatic cells are still to be elucidated. The earliest evidence for DNA repair in mammalian oocytes comes from work in the 1970s, where it was shown that oocytes can synthesize DNA in response to DNA lesions. In these studies, it was also shown that GV oocytes arrested at prophase have a better capacity for synthesizing new DNA compared to MI and MII oocytes [32,33,34]. Specifically in regard to DSB repair, characteristic assays to detect DNA repair include the determination of γH2AX (a DSB marker) localization over time and the comet assay. It has been shown that over a time course of up to 10 h after treatment of GV-arrested mouse oocytes with etoposide, the cells show a gradually decreasing staining for γH2AX, indicating that DNA damage is being repaired [30]. Similar results have been shown with the use of Doxorubicin prior to performing comet assays, where the length of the comet tail decreases over time [35].

During GV prophase arrest, oocytes have already duplicated their DNA, therefore HR seems like a good candidate for the repair of DSBs since sister chromatids are present and attached to each other [36]. Evidence supporting the presence of a possible HR-dependent mechanism for DNA repair come from oocytes treated with genotoxic agents, such as etoposide. Mouse GV oocytes treated with etoposide and lacking Ooep, a regulator of Rad51 and ATM, show no apparent DNA repair-related reduction in γH2AX staining [37]. Furthermore, in etoposide-treated post-GVBD oocytes, inhibition of Rad51, through the use of the Rad51 inhibitor RI-1, resulted in reduced incorporation of EdU in the DNA undergoing repair [38]. This report also suggests the existence of a Rad52-dependent DNA repair mechanism in fully grown mouse oocytes, since Rad52 seems to colocalize with γH2AX following DNA damage.

HR repair has also been examined in the AKR/J mouse strain. DSBs seem to be a feature of oocytes of inbred strains of mice, such as AKR/J and the maintenance of these DNA breaks leads to oocyte death. However, when oocytes from AKR/J mice are microinjected with a recombinant form of Rad51, a crucial mediator of HR, oocyte death is significantly reduced [39]. In addition, overexpression of Rad51 in fully grown mouse oocytes led to a reduction in the rate of cell death following incubation with Doxorubicin [35]. Similar observations are also seen in bovine oocytes. Irradiation with krypton-78 or UV-B of MII bovine oocytes led to the partial activation of a cell death program. However, the introduction of recombinant Rad51 into the oocytes reduced the levels of DNA damage and alleviated the cell death-related processes [40]. Therefore, HR might potentially be induced following DNA damage, but it is not clear whether HR is sensitive or efficient in mammalian oocytes.

In another report, it is suggested that melatonin may enhance NHEJ-dependent repair processes in GV-stage mouse oocytes [41]. Another hypothesis implicates actin filaments in the DNA repair processes. In one report, the authors suggest that the cumulus cells attached to the oocyte induce the production of nuclear actin filaments [42] which are known to participate in DDR processes [43]. This hypothesis is supported by the fact that an actin nucleation protein, JMY, translocates to the nucleus upon etoposide-induced DNA damage [44].

Maternal age may play a crucial role in the DNA repair capacity. Aged human [45] and mouse [46] oocytes are reported to show increased DNA damage with advanced age. In addition, the repair capacity might be compromised as important proteins (ATM, Rad51, Mre11, BRCA1) seem to be downregulated in aged human and mouse oocytes [46]. Therefore, one aged oocyte feature leading to age-dependent infertility may be a reduced efficiency of DNA repair processes.

## 6. The DNA Damage Response in MII Oocytes

There are also indications that aspects of DDR mechanisms exist in the female gamete, the MII-arrested oocyte, before and after fertilization. When MII-arrested bovine oocytes are exposed to UV irradiation, egg activation is impaired. Following fertilization, a male pronucleus is formed normally, but the female pronucleus is abnormal or absent [14]. There are also reports regarding the function of the BER pathway in oocytes. BER components, such as APE1 and XRCC1, are expressed in mouse MII oocytes. Upon fertilization, OGG1, a DNA glycosylase crucial for BER, becomes active following post-translational modifications. This increases excision of a specific DNA lesion 8OHdG, revealing a possible synergy between the female and male gametes against oxidation damage [47].

Recent studies also indicate the potential participation of NHEJ in the repair of DNA damage in MII oocytes [48]. MII mouse oocytes treated with high concentrations of etoposide showed slower DNA repair, as monitored by the persistence of γH2AX DNA staining, following inhibition of DNA-PK or DNA ligase IV [48]. Furthermore, the plasma membrane seems to aid the MII oocyte’s response to potential genotoxic stress. Although following etoposide treatment MII-arrested oocytes show strong γH2AX staining on the metaphase plate, fertilized oocytes treated with etoposide do not. This does not seem to be the result of the inactivation of DDR mechanisms with fertilization. Instead, it is related to changes in the permeability of the plasma membrane. Following fertilization, permeability glycoprotein (PGP) becomes associated with the oocyte plasma membrane and mediates the efflux of etoposide [49]. Therefore, the fertilized oocyte becomes protected from soluble genotoxic agents by minimizing their presence in the cytoplasm.

The oocyte can also support a DDR response for DNA damage in the spermatozoon DNA. Sperm DNA can potentially harbor DSBs or other DNA damage [50]. The oocyte’s machinery becomes active following fertilization and the formation of the zygote to respond to sperm DNA damage in the male pronucleus. This response seems to be regulated by a p53-dependent S-phase DNA damage checkpoint [51,52].

## 7. Physiological Role of DDR Components in Oocytes

Many DDR factors and components exhibit non-DDR functions during meiosis in fully grown oocytes (Table 2).

BRCA1 has been found to localize at spindle poles in MI oocytes in the absence of DNA damage. Following spindle disruption, after treatment with taxol, BRCA1 localizes on spindle fibers and on cytoplasmic asters, while treatment with nocodazole leads to BRCA1 localization on chromosomal sites. Disrupting BRCA1 through an inhibitory antibody or RNAi leads to the formation of abnormal spindles with many misaligned chromosomes and the dissociation of γ-tubulin from spindle poles. Surprisingly however, the SAC does not seem to become active in BRCA1-depleted oocytes and MAD2L1 is not recruited to kinetochores. As a result, BRCA1-depleted oocytes do not arrest at MI. Therefore, BRCA1 might be important for both spindle integrity and SAC establishment in mammalian fully grown oocytes [53].

Another DNA repair factor, whose disfunction is linked to breast cancer, BRCA2, also seems to be involved with oocyte physiology. Follicle development in BRCA2-deficient mice is dysregulated and female mice are infertile. Fully grown oocytes from these mice rarely reach the MII stage, and instead remain arrested in MI with spindle defects and misaligned chromosomes [54]. In MII oocytes and zygotes, BRCA2 seems to bind to the proinsulin-derived C-peptide. Although the role of this interactions is not determined, it might explain the meiotic disfunctions and infertility observed in diabetic women [55].

Chk1 also seems to have a role during mammalian oocyte maturation. In mouse and pig oocytes, Chk1 plays a part in meiotic resumption and depletion of Chk1 by siRNA or chemical inhibition leads to elevated rates of GVBD. In the absence of Chk1, Cdc25A becomes upregulated. In addition, the APC/C coactivator Cdh1 is reduced, and this allows for the accumulation of Cyclin B. Chk1 overexpression in GV oocytes impairs meiotic resumption and GVBD is inhibited [56,57]. Chk1 overexpression following GVBD in mouse oocytes activates the SAC and reduces the rate of Pb1 extrusion. However, it is not clear if Chk1 acts directly on the SAC, or indirectly by acting on spindle formation [56]. In pig oocytes, Chk1 knockdown leads to meiotic arrest in MI and severe chromosome misalignments [57]. On the other hand, Chk2 inhibition with a specific chemical inhibitor resulted in prolonged GV arrest, which was rescued after removal of the inhibitor. Similarly to Chk1, Chk2 inhibition following GVBD led to a reduced rate of Pb1 extrusion, possibly due to the observed spindle and chromosomal disruption [58].

There are indications that the HR component, Rad51, plays a role in the physiological process of oocyte maturation and the progression of MI. RNAi for Rad51 in mouse oocytes leads to MI arrest due to spindle and chromosome anomalies. The role of Rad51 in MI may be linked to mitochondrial integrity, since Rad51 depletion disrupted mitochondrial number, function and distribution [59]. In a separate report, the pharmacological inhibition of Rad51 in pig oocytes led to elevated levels of DNA damage, impairment of oocyte maturation, spindle defects and dysregulation of mitochondrial activity [60].

Another DSB repair protein, the tumor suppressor p53-binding protein 1 (TP53BP1), also seems to affect spindle formation. RNAi for TP53BP1 impairs oocyte maturation by destabilizing microtubule organizing centers (MTOC) which causes the formation of nonbipolar spindles and chromosome misalignment [61].

It must be noted that phosphorylation of H2AX at serine 139 (γH2AX) occurs physiologically during resumption of meiosis in mammalian oocytes [16]. It is hypothesized that ATR and Mre11 may be responsible for H2AX phosphorylation in the absence of DNA damage; however, the purpose of this phosphorylation is not yet clear [16]. Nevertheless, chromosome integrity in MI seems to depend on the actions of Mre11, since Mre11 inhibition by mirin significantly increased chromosome segregation errors in mouse oocytes [16].

**Table 2:** Physiological roles of DDR proteins in oocytes. The names of genes and their proteins are in accordance with Uniprot entries. DDR: DNA damage response, DSB: double-strand break, HR: homologous recombination, NHEJ: nonhomologous end joining, ssDNA: single-stranded DNA, GVBD: germinal vesicle breakdown, MI: meiosis I, MII: meiosis II, SAC: spindle assembly checkpoint, MTOC: microtubule organizing center.

Organism	Gene	Protein	Role in DDR	Expression during Maturation	Subcellular Localization during Maturation	Role in Oocyte Physiology	Reference
*Mus musculus* (Mouse)	Brca1	Breast cancer type 1 susceptibility protein homolog	Promoting HR and antagonizes 53BP1 [62]	Low at GV, increases after GVBD, max at MI and stable afterwards	Germinal Vesicle at GV stage, after GVBD near chromosomes and after prometaphase I at spindle poles. At anaphase I was localized at the midbody and then spindle poles again in MII	Role in spindle assembly, chromosome alignment and SAC regulation	[53]
*Mus musculus* (Mouse)	Chek1	Serine/threonine-protein kinase Chk1	Involved in cell cycle arrest (intra-S G2/M), repair of damaged DNA [63]	Steady expression during maturation from GV to MII stage	Germinal vesicle and after GVBD at the spindle poles	Essential for the GV arrest of oocytes. Involved in the regulation of SAC	[56]
*Sus scrofa* (Pig)	CHEK1	Checkpoint kinase 1	Involved in cell cycle arrest (intra-S, G2), repair of damaged DNA [63]	Steady expression which reaches max levels at MI	Cytoplasm and after GVBD at the spindle	Involved in the regulation of Cyclin B-CDK1 and SAC in order for MI to be successful	[57]
*Mus musculus* (Mouse)	Chek2	Serine/threonine-protein kinase Chk2	Involved in cell cycle arrest (G1/S G2/M), repair of damaged DNA, apoptosis [63]	-	GV→centromeres→spindle poles	Plays roles in maintaining GV arrest and entry in GVBD and spindle assembly, chromosome alignment and SAC	[58]
*Mus musculus* (Mouse)	Rad51	DNA repair protein RAD51 homolog 1	Strand invasion during HR [64]	Steady expression until MI then decreases	-	Plays pivotal role in mitochondrial, spindle and chromosomal integrity	[59]
***Sus scrofa*** **(Pig)**	pigRad51	DNA repair protein RAD51 homolog	Strand invasion during HR [64]	Reduction after GVBD and then increase in MII	GV and then cytoplasm	Involved in completion of MI. Roles in mitochondrial integrity. Spindle formation, chromosomal alignment, DNA damage repair	[60]
***Mus musculus (Mouse)***	Tp53bp1	TP53-binding protein 1	Promoted NHEJ. Antagonizes with BRCA1 [65]	-	Localized like a cloud around DNA/spindle	Important in spindle bipolarity and MTOC and chromosome alignment	[61]
***Mus musculus (Mouse)***	Brca2	Breast cancer type 2 susceptibility protein homolog	Loading of Rad51 to ssDNA during HR [64]	-	Possible cytoplasmic localization in MII oocytes. Following fertilization, BRCA2 shows peri-pronuclear localization	Oocyte maturation, spindle assembly, chromosome alignment	[54,55]
*Mus musculus* (Mouse)	Mre11	Meiotic Recombination 11	Sensing DSBs, role in repair [66]	GV stage, MI	Nuclear localization in GV-stage oocytes	Chromosome integrity	[16]
*Mus musculus* (Mouse)	H2AX	Histone H2AX	Phosphorylation of H2AX at Ser-139 (γH2AX) is important for signaling and initiating the repair of DSBs [48]	MI, MII	γH2AX localization on the entire chromosome	Unknown	[16]

## 8. Omics and the Oocyte DDR

Important insight into the potential factors and mechanisms involved in the mammalian oocyte DDR can be provided by the use of omic technologies, such as proteomic and transcriptomic analyses. Indeed, omic analyses provide valuable information regarding the presence of DDR mechanisms in mammalian oocytes. Martin et al. present in detail specific DDR factors identified through omic technologies [55].

Several studies have examined the proteome and transcriptome of mouse, monkey or human oocytes at different stages of oocyte maturation and show that oocytes possess a plethora of DDR proteins and transcripts that code for DDR proteins [67,68,69,70,71]. This is not surprising because of the transcriptionally inactive nature of the fully grown mammalian oocyte and the fact that, for its functions, the oocyte relies predominantly on pools of mRNAs or dormant protein complexes.

More specifically, proteomic analyses show that more than 40 factors involved in DNA repair mechanisms, such as BER, nucleotide-excision repair (NER), HR and NHEJ, are present in mammalian oocytes. These analyses also reveal that repair proteins are present during all stages of mouse oocyte maturation, being more abundant at the MII stage [72]. DDR factor abundance in MII oocytes, compared to GVs, may represent a robust repair machinery at the MII stage or following fertilization. However, this remains to be verified experimentally.

Transcriptomic analyses show that oocytes possess more than 100 transcripts that code for factors involved in DDR mechanisms, such as BER, NER, mismatch repair (MMR), HR and NHEJ. It is interesting that a great proportion of these transcripts are more abundant in the MII oocyte than in blastocysts [73]. However, this may not necessarily represent more robust DDR mechanisms in MII oocytes. Unlike the MII oocyte, the somatic cells of the blastocyst are capable of transcription, and therefore it may not be required that the blastocyst stem cells maintain extensive stores of dormant transcripts, such as the ones detected in oocytes. Another finding identified from transcriptomic analyses is that there seems to be selective degradation of transcripts related to DNA repair (118 different transcripts) from the GV to the MII stage [74]. However, this transcript expression profile does not necessarily correspond to protein expression and function.

An important level of gene expression regulation involves miRNAs. A study in 2015 analyzed human oocyte and blastocyst microarray data in combination with microRNA databases and evaluated a potential relationship between 10 mRNAs that code for DNA damage repair factors and 20 miRNAs known to target repair and checkpoint genes. This was the first indication that several DDR-related miRNAs are present in oocytes. Interestingly, higher expression of miRNAs was found in oocytes compared to blastocysts. However, this is a general trend in oocyte miRNAs and may represent a mechanism by which oocytes silence their large pool of mRNAs. This report suggests specific direct or indirect, stabilizing or destabilizing, relationships between miRNAs and mRNAs. Some miRNAs seem to have the potential of stabilizing MMR mRNAs in the oocyte, allowing this repair pathway to be active from the oocyte to the preimplantation stage of development. On the contrary, other miRNAs seem to impose a destabilizing effect on mRNAs corresponding to DNA repair genes involved in NER, ICL repair or DSB repair, indicating a potential insensitivity of these mechanisms in oocytes [75].

In addition, gene expression assays reveal that reproductive age may influence the expression of several DDR genes. In aged human oocytes, more than 20 mRNAs coding for checkpoint and repair genes, involved in BER, NER and DSB repair, show reduced expression compared to oocytes from young individuals. These data indicate a potential dysfunction of DDR mechanisms in aged oocytes [46,76,77].

## 9. Conclusions

DNA damage in mammalian oocytes can potentially introduce genomic instability and mutations to the future embryo. Therefore, it is imperative to understand whether oocytes respond to DNA damage and what DDR mechanisms are active in order to prevent genomic damage to be transferred to the embryo. Over the past decade, scientists have examined the existence and function of DDR mechanisms in mammalian oocytes. There is general consensus that the fully grown oocyte cannot launch a robust DNA damage checkpoint. However, a lot of work needs to be done to elucidate the DNA repair pathways that function in mammalian oocytes.

## Figures and Tables

**Table 1:** Characteristic proteins involved in the DNA damage checkpoints. It is worth noting that some proteins belong to more than one category (for example ATM kinase is a sensor and a signal transducer) [3,4].

Sensors	Mediators	Transducers	Effectors
Rad9-Rad1-Hus1 (9-1-1 complex) Rad17-RFC_2-5_ complex Mre11-Rad50-NBS1 (MRN complex) ATM ATR-ATRIP	BRCA1 53BP1 TopBP1 MDC1 Claspin	Chk1 Chk2	p53 Cdc25 phosphatases CDC7 Cyclin-CDKs complexes

## Data Availability

Not applicable.
